# Biomass derived carbon/platinum nanoparticles as electrocatalyst for the hydrogen evolution

**DOI:** 10.1038/s41598-024-84727-z

**Published:** 2025-02-07

**Authors:** Shimaa M. Ali

**Affiliations:** Chemistry Department, Faculty of Science, Cairo University, P.O. 12613, Giza, Egypt

**Keywords:** Catalysis, Electrochemistry

## Abstract

Green hydrogen is gaining a significant attention in the transition to sustainable energy and achieving net-zero emissions. Platinum-based catalysts are highly regarded in hydrogen production, particularly due to their efficiency in water electrolysis. Platinum nanoparticles (Pt NPs) is successively prepared by the microwave-assistant citrate method on a biomass-based support, and characterized by X-rays diffraction, scanning and transmission electron microscopy. The chelation and gelation resulted by using citric acid during the synthesis lead to the formation of highly stabilized and dispersed Pt NPs on the carbon support. The electrocatalytic activity of Pt NPs for the hydrogen evolution reaction (HER) is examined by cathodic linear polarization and impedance spectroscopy. A high catalytic performance is shown by the prepared sample, as indicated by the calculated exchange current density 5.3 mA/cm^2^, and activation energy, 38.13 kJ/mol. The HER follows Volmer/Tafel mechanism with a reaction order of unity. Impedance spectra confirms the high electrocatalytic activity by the decrease of the total impedance, pore resistance, and charge-transfer resistance, with increasing the applied overpotential. The proposed synthesis method offers a green, economic, and efficient route for preparing precious metals used for catalytic applications.

## Introduction

The global energy crisis and environmental pollution caused by fossil energy have generated interest in the development of alternative sources of energy. This has led to the search for renewable energy sources that are abundant, cost-effective, environmentally friendly, and have a balance between supply and storage^1–9^. Over the past ten years, there has been increasing interest in the production of hydrogen gas as a source of clean fuel. To be economically viable, hydrogen gas must be produced in an energy efficient manner. For this reason, there has been an increased interest in the development of highly active and efficient low-cost electrocatalysts to reduce the overpotential needed to drive the hydrogen evolution reaction in acidic solutions^10–14^. Platinum-based electrocatalysts have garnered extensive attention due to their high activity and stability toward the hydrogen evolution reaction (HER). However, the high cost and scarcity of platinum, combined with overpotential and slow kinetics, limit the practical applications of the aforementioned electrocatalysts. To resolve this problems, significant efforts are focused on amplifying the activity and lowering the loading amount of the noble metal with design and synthesis strategies for novel electrocatalysts^15–18^.

The ability to control the synthesis of nanoparticles with respect to their size, polydispersity, and morphology is of paramount importance in obtaining nanostructured materials with the desired properties. Among the numerous methods for the synthesis of metal and metal oxide nanoparticles, citrate-based chemical reduction methods have been historically successful, economical, and reproducible approaches using citric acid as both a reducing and stabilizing agent^19–21^. For the synthesis of platinum nanoparticles (Pt NPs), citric acid is a biocompatible and naturally sourced stabilizing agent. Pt NPs were prepared via a modified polyol method, which utilizes ethylene glycol as an essential reducing and dispersing agent, and citric acid as an additional precursor for stabilization^22,23^. Different molar ratios of citric acid and Pt salt precursor (H_2_PtCl_6_) were investigated. Pt NPs were then characterized by UV-vis spectroscopy and dynamic light scattering (DLS) analysis, showing that citric acid was effective for stabilizing Pt NPs as no agglomeration was observed. Pt NPs stabilized by citric acid were incorporated into carbon supports, and the resultant electrocatalysts were investigated for the HER. A synergistic effect between Pt NPs and carbon supports was observed, resulting in a more efficient transition metal (TM)-free catalyst for the HER. Among the various carbon supports, nitrogen-doped graphene oxide (NGO) exhibited the highest catalytic efficiency^22,23^. Electrochemical investigations of the NGO-supported Pt NPs revealed that citric acid-stabilized Pt NPs exhibited higher catalytic efficiency than their ethylene glycol-stabilized counterparts. Overall, Pt NPs synthesized with citric acid were most favorable in terms of size, uniformity, and electrocatalytic activity toward the HER. Metal citrate is thought to be a good candidate to both provide metal precursors and promote a more homogeneous dispersion of metal nanoparticles due to its metal transplantation and co-chelation capability^24^. It can be expected that metal nanoparticles would form as a consequence of metal citrate decomposition, and these nanoparticles would be well electrolyzed in the carbonaceous materials derived from the precursors. Chelated metal citrate could promote homogeneous dispersion of metal ions in the gel during the pre-heating step and yield well-dispersed metal nanoparticles in the silica matrices upon the thermal decomposition^25,26^. A well-dispersed Pt NPs supported on rhodia is prepared by citrate-assisted method and applied as an electrocatalyst for the CO conversion at lower reduction temperature^27^. The high electrocatalytic activity is due to the stabilized and dispersed Pt NPs on the support which discussed based on the following mechanism; Pt-citrate layer is formed, followed by chelation. While attaining a solvent evaporation and complete drying, the viscosity increased by gelation and isolate; stabilize, Pt particles from each other. It also inhibits the migration to the support edges, resulting in a highly distributed NPs over the support^28–32^.

In this work, Pt NPs will be prepared by the microwave-assisted citrate method on a biomass support, derived from banana peels, the latter offer a green and efficient solution to the waste management, as well as, massive production of the nanomaterials^33–35^. The microwave-assisted method for preparing nanomaterials offers a rapid heating, energy efficiency, controlled size of a narrow distribution and enhanced properties^36–38^. The synthesized NPs will be characterized by X-rays diffraction (XRD), scanning and transmission electron microscopes (SEM and TEM), and BET specific surface area measurements. The electrocatalytic activity for the HER in acidic medium will be tested and evaluated by electrochemical techniques; cathodic linear polarization and electrochemical impedance spectroscopy (EIS). A kinetic study will be made to deduce the mechanism of the catalyst performance.

## Results and discussion

### Structural and surface characterization of pt NPs

**Fig. 1:**
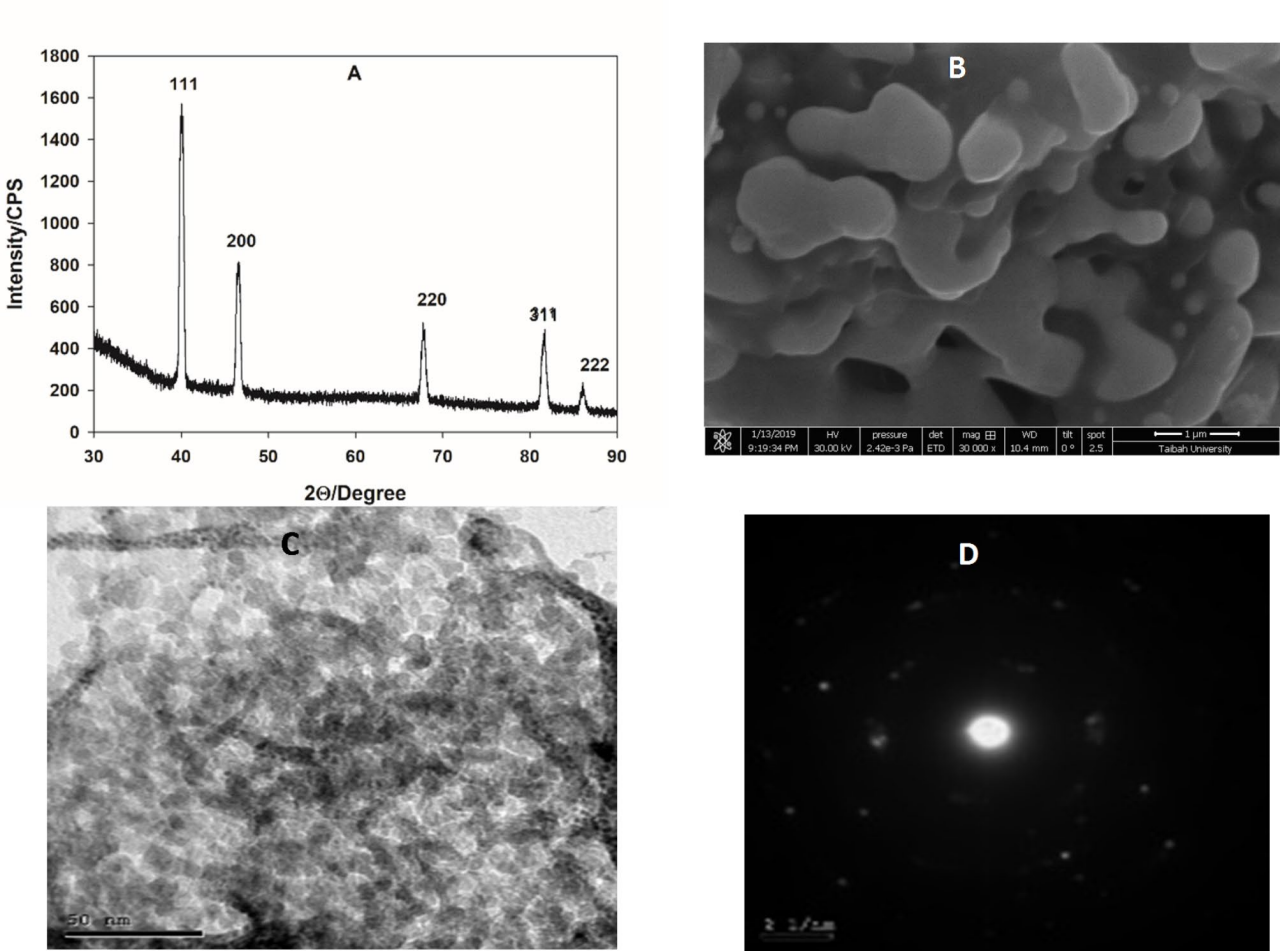
(**A**) XRD, (**B**) SEM, (**C**) TEM, and (**D**) SAED of Pt NPs prepared by the microwave-assisted citrate method.

The structural composition of Pt NPs, prepared by microwave-assisted citrate method, is identified by XRD. Figure 1A represents the XRD pattern of the prepared sample, data are indexed and compared with the COD card of cubic platinum, card number: 1,011,107. Results show that the sample is composed of a pure single phase of cubic platinum as indicated by the appearance of five characteristic peaks (111), (200), (220), (311), and (222), respectively. The particle size (τ) can be calculated by Scherrer equation^39^:

1 $$\tau\:\:=\frac{0.9\:\lambda\:}{\beta\:\:cos\:\theta}$$

where τ is the particle size (nm), λ is the wavelength of incident X-ray (CuK_α_ = 0.154 nm), β is the full width at half maximum, and θ is the Bragg’s angle.

The average particle size equals 3.1 nm, in addition, the lattice parameter, *a*, lattice volume, *V*, and the theoretical density *D*_*theo*_ are calculated according to the following equation:

2 $$\:\frac{1}{{d}^{2}}=\frac{{h}^{2}+{k}^{2}+{l}^{2}}{{a}^{2}}$$

3 $$\:V={a}^{3}$$

4 $$\:{D}_{theo=\frac{Z\:\times\:\:M}{{N}_{A}\:\times\:\:V}}$$

here, *d* is the space between diffracting planes, *h*, *k*, and *l* are miller indices, *Z* is a constant = 4, *M* is the molecular weight of the sample = 195.08 g/mol, *N*_*A*_ is Avogadro’s number. The calculate parameter are; *a* = 3.911 Å, *V* = 59.82 Å^3^, and *D*_*theo*_ = 19.96 g/cm^3^.

The morphology of the prepared Pt NPs is examined by SEM and TEM, as shown in Fig. 1B,C, respectively. SEM photo revels interconnected distorted cubic grains of a high porosity. While, TEM image shows that the Pt NPs are highly and homogeneously distributed particles on the carbon support. The average size is 4.2 nm, close to the value calculated by Scherrer’s equation. Figure 1D represents a selected area electron diffractogram (SAED) of the sample, which indicated the polycrystallinity of Pt NPs. The BET specific surface area of the prepared Pt NPs equals 63.16 m^2^/g.

### The electrocatalytic activity for the HER

**Fig. 2:**
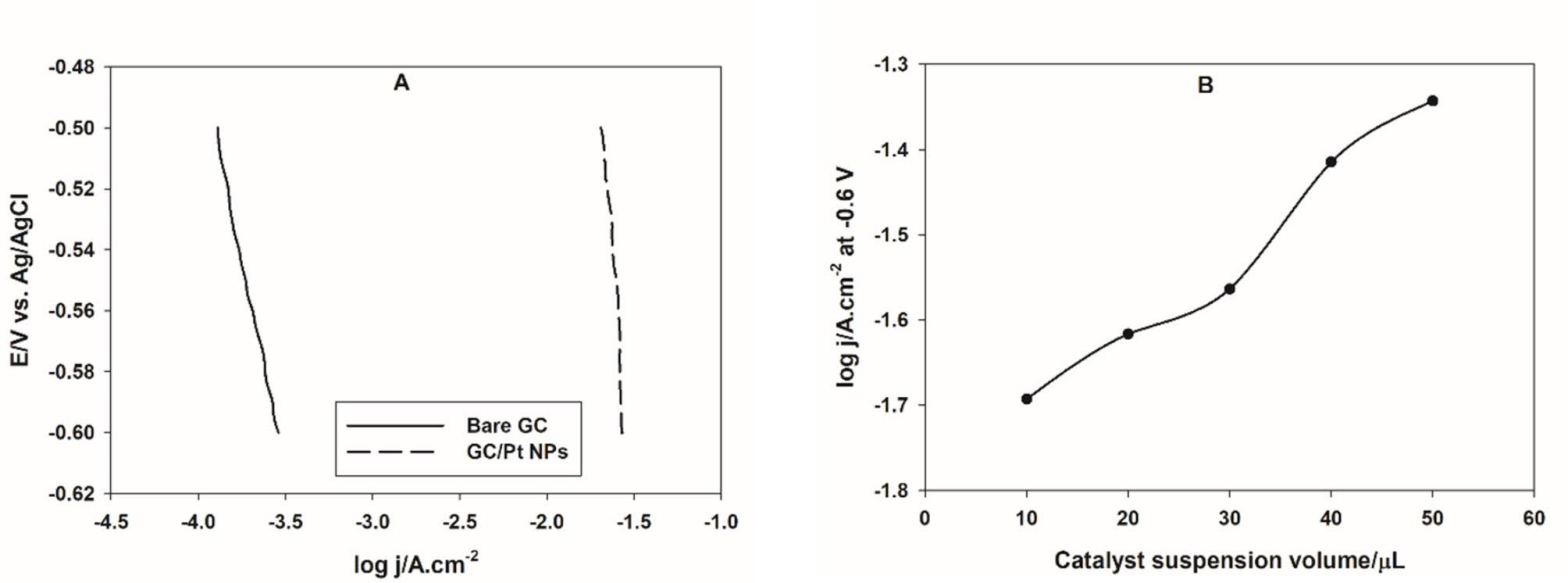
(**A**) Cathodic linear polarization curves of bare GC and CG electrode casted with 30 µL of Pt NPs in 0.1 M H_2_SO_4_ solution. (**B**) the variation of the HER rate with Pt NPs loading.

To examine the electrocatalytic activity of Pt NPs, a cathodic linear polarization experiment is performed in 0.1 M H_2_SO_4_ solution, the data is analyzed and important electrochemical parameters, such as Tafel slope, *b*, and exchange current density *i*_*o*,_ are calculated. Figure 2A shows a comparison between the potentiodynamic polarization lines of a bare GC and Pt NPs casted on GC electrode in 0.1 M H_2_SO_4_ solution. It is clear that the presence of Pt NPs catalyst enhances the electrocatalytic activity for the HER by about 100 times. This indicated the increased catalytic active sites by casting Pt NPs on GC electrode. The excellent electrocatalytic activity is attributed to the homogeneous dispersion of metal ions in the chelated metal citrate gel, which yields well-dispersed and stabilized metal nanoparticles in the support upon the thermal decomposition.

According to Tafel equation^39^:

5 $$\:\eta\:=a-b\text{log}j$$

where, *η* is the applied overpotential in V, *j* is the current density in A/cm^2^, *a* and *b* are Tafel intercept and slope, respectively, which can be expressed by the following equations:

6 $$\:a=\:\frac{2.303RT\text{log}{i}_{o}}{\beta\:nF}$$

7 $$\:b=\:\frac{2.303RT}{\beta\:nF}$$

where *i*_*o*_ is the exchange current density (A/cm^2^), represents the rate of the HER at equilibrium, R is the universal gas constant (8.314 J/mol K), T is the absolute temperature in K, β is the symmetry factor, n is the number of electrons, and F is Faraday’s constant (96,485 C/mol).

The value of the Tafel slope, in the presence of Pt NPs, is 807.2 mV/dec. The Tafel slope provides insights into the rate-determining step of the HER. For Pt, the HER typically involves the Volmer step (proton discharge; Eq. 8), the Heyrovsky step (electrochemical desorption, Eq. 9), and the Tafel step (recombination of adsorbed hydrogen atoms, Eq. 10). A high Tafel slope might suggest that the Tafel step is the rate-determining step^40,41^. In some cases, the structure and morphology of the Pt catalyst can significantly influence the Tafel slope. For instance, locally trapped hydrogen in the catalyst layer can lead to inconsistencies in the Tafel slope measurements^42^.

8 $${\text{M}}+{{\text{H}}_{\text{3}}}{{\text{O}}^+}+{{\text{e}}^ - } \to {\text{M}}{{\text{H}}_{{\text{ads}}}}+{{\text{H}}_{\text{2}}}{\text{O}}$$

9 $${\text{M}}{{\text{H}}_{{\text{ads}}}}+{{\text{H}}_{\text{3}}}{{\text{O}}^+}+{{\text{e}}^ - } \to {{\text{H}}_{\text{2}}}+{\text{M}}+{{\text{H}}_{\text{2}}}{\text{O}}$$

10 $${\text{MH}}_{\text{ads}}+{\text{MH}}_{\text{ads}} \to {\text{H}}_{2}+2{\text{M}}$$

The exchange current density *i*_*o*,_ for the HER is a critical parameter that indicates the intrinsic activity of the catalyst. For the prepared Pt NPs, the calculated *i*_*o*_ equals 5.3 mA/cm^2^. This value is higher than that reported for Pt(111) (0.45 mA/cm^2^) and other transition metals in H_2_SO_4_ medium (Co and Ni, 3.6 and 2.6 µA/cm^2^, respectively) suggesting the excellent electrocatalytic activity of the Pt NPs prepared by the microwave-assisted citrate method^43^.

The catalyst loading is optimized by performing the polarization experiment of GC electrode casted with different microliters of Pt NPs suspension in 0.1 M H_2_SO_4_ solution. Figure 2B shows the variation of the rate of the HER, expressed as *log j* at a considerable potential of -0.6 V, with the catalyst loading, expressed as µLs of Pt NPs suspension casted on GC electrode. It can be seen that the rate of the HER continues to increase with increasing the catalyst loading. This is primarily because a higher catalyst loading increases the number of active sites available for the reaction, thereby facilitating more hydrogen production^44,45^. However, the optimal catalyst loading in the next sections, is taken to be 30 µL.

### Kinetics study

**Fig. 3:**
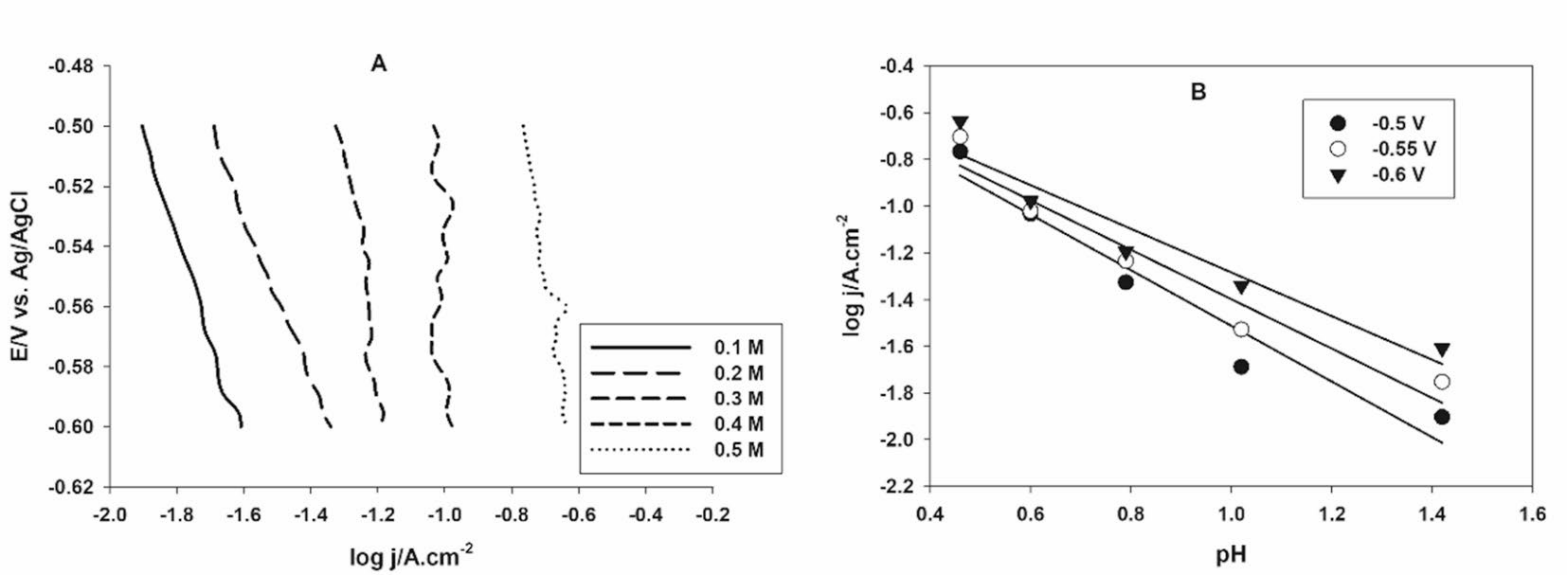
(**A**) Cathodic linear polarization curves of CG electrode casted with 30 µL of Pt NPs in different concentrations of H_2_SO_4_ solutions. (**B**) The dependence of the HER rate at different overpotentials on the pH.

Kinetics of the HER can be studied by performing the potentiodynamic polarization of GC electrode casted with 30 µL of Pt NPs suspension in different concentrations of H_2_SO_4_ solutions at a constant ionic strength, the latter is kept constant by adding Na_2_SO_4_. Figure 3A shows the polarization curves of GC/Pt NPs in x M H_2_SO_4_ solutions, x = 0.1, 0.2, 0.3. 0.4, and 0.5 M. The rate of the HER increases with increasing the acid concentration, the reaction order can be calculated from the slope of the HER rate vs. the H_2_SO_4_ solution pH values, as shown in Fig. 3B. The average reaction order, calculated at three different overpotentials, is 1.06. The reaction order is often found to be close to 1, indicating that the rate-determining step involves a single proton transfer, for the proposed catalyst the HER follows Volmer-Tafel mechanism^46,47^.

### Determination of the activation energy

**Fig. 4:**
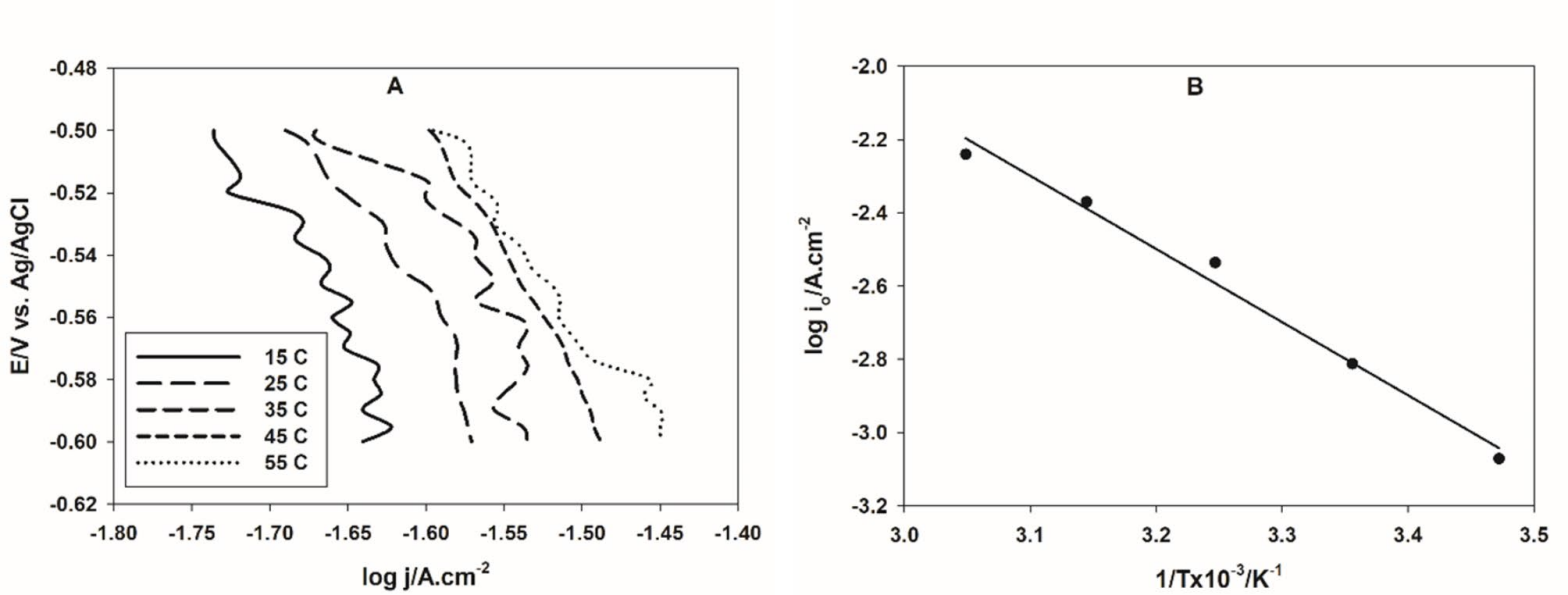
(**A**) Cathodic linear polarization curves of CG electrode casted with 30 µL of Pt NPs in 0.1 M H_2_SO_4_ solution at different temperatures. (**B**) The Arrhenius plot.

Figure 4A shows the potentiodynamic polarization of GC electrode casted with 30 µL of Pt NPs suspension in 0.1 M H_2_SO_4_ solution at different temperatures. The rate of the HER increases with the temperature rise. The activation energy, *E*_*a*_, can be calculated according to Arrhenius equation^48^:

11 $$\:\text{log\:}{i}_{o}=\text{log}A\:-\:\frac{{E}_{a}}{2.303RT}$$

where A is the pre-exponential constant, related to the entropy change of the reaction. Figure 4B shows Arrhenius plot, the calculated *E*_*a*_ value for the HER on Pt NPs prepared by the microwave-assisted citrate method is 38.13 kJ/mol. This value is smaller than most of Pt-based catalysts reported in literature, as shown in Table 1, reflecting the enhanced catalytic propertied of Pt catalyst prepared by the proposed method in this work.

**Table 1:** A comparison of the *E*_*a*_ values of the HER on reported Pt-based catalysts.

Catalyst	E_a_ (kJ/mol)	References
PtNi@SiO2	54.76	^49^
Pt-CoCu@SiO2	51.01	^50^
NiCo-Pt NPs	45.72	^51^
Pt0.65Ni0.35	39.01	^52^
PdPt sNPs	57.30	^53^
Pt(8%)/CCF-500	39.2	^54^
Pt NPs/C	38.13	This work

### Electrochemical impedance spectroscopy (EIS)

**Fig. 5:**
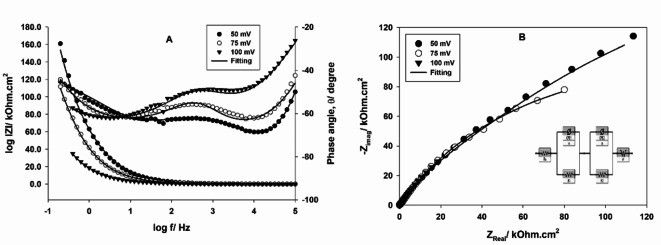
(**A**) Bode, (**B**) Nyquist plots of GC/Pt NPs, prepared by the microwave-assisted citrate method, in 0.1 M H_2_SO_4_ solution at different overpotentials, the inset represents the electrical equivalent circuit used for fitting.

To better understanding of the HER mechanism, EIS spectra of GC casted with Pt NPs, prepared by the microwave-assisted citrate method are recorded in 0.1 m H_2_SO_4_ at different overpotentials of 50, 75, and 100 mV. Bode and Nyquist plots are displayed in Fig. 5A,B, respectively. Data are analyzed and fitted to the electrochemical equivalent circuit (EEC), shown in Fig. 5B, inset^55^. There is a good agreement between experimental (symbols) and fitted (lines) data, suggesting the suitability of the proposed EEC. According to EIS spectra, there are two-time constants; *CPE1* and *CPE2*, related to the kinetics of the HER and electrode porosity, respectively ^55^. By increasing the applied overpotential, In the Bode plot, the value of the total impedance at low frequency is decreased, and the value of the phase angle shifts to less negative value due to the decreased pore resistance^56^. This reflects the good catalytic activity of Pt NPs. On the other hand, Nyquist plot consists of two semi-circles and 45° linear part at low frequencies. The radius of the first semicircle, at high frequencies, remains constant by increasing the applied overpotentials, because the electrode porosity is not affected. While, the radius of the second semicircle, at intermediate frequencies, decreases with increasing the applied overpotentials, reflecting the decreased charge-transfer resistance and indicating the high electrocatalytic activity of the proposed catalyst. Electrochemical parameters of the EEC are calculated by the fitting and listed in Table 2. With increasing the overpotentials, values of resistances R_1_ and R_2_ are decreased, the value of CPE 1 increases, while that of CPE 2 is independent, and the value of the Warburg component, diffusion of protons, is decreased.

**Table 2:** Electrochemical parameters of the EEC, calculated by the fitting.

E/mV	*R*_s_/Ω cm^2^	CPE1/µF/cm^2^	*n*	*R*_1_/kΩ cm^2^	CPE2/µF/cm^2^	m	*R*_2_/k Ω cm^2^	W/Ω/s^½^
− 50	2.91	7.39	0.7249	340.04	15.49	0.7411	456.61	54.57
− 75	3.65	13.19	0.8437	275.91	15.96	0.7235	279.80	46.41
− 100	3.35	24.98	0.7163	129.90	14.59	0.7347	236.31	37.81

### Stability of the catalyst

**Fig. 6:**
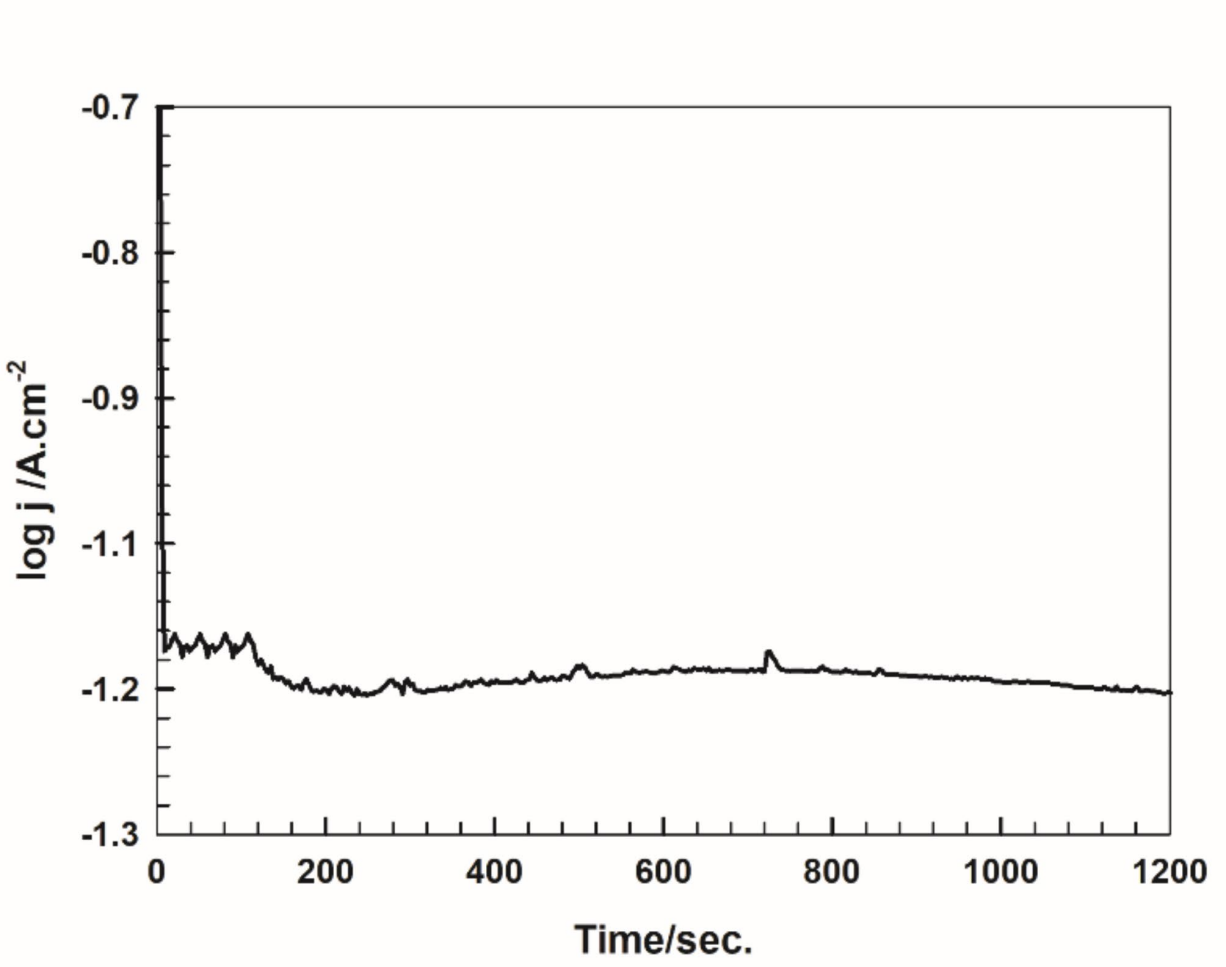
The current-time response of Pt NPs, prepared by the microwave-assisted citrate method, in 0.1 M H_2_SO_4_ solution at − 0.5 V.

The stability of the prepared Pt NPs for the HER is examined by recording the current-time response in 0.1 M H_2_SO_4_ solution at − 0.5 V, for 20 min, as shown in Fig. 6. The current is almost constant (% decrease in current is less than 5%), reflecting the good operation stability under potentiostatic conditions.

## Experimental

### Materials

Platinum (II) nitrate (99.95%, Glentham), citric acid (99%, Aldrich), nitric acid (70%, A.C.S. reagent, Aldrich), sulfuric acid (95–98%, Aldrich), calcium hydroxide (ACS reagent, ≥ 95.0%, Sigma-Aldrich), and dimethyl formamide (DMF) (anhydrous, 99.8%, Sigma-Aldrich) used as received. All solutions are prepared by double distilled water. All electrochemical measurements are performed in oxygen-free solution, which can be achieved by a continuous nitrogen gas (99.99%) bubbling inside the solution for 10 min prior to the experiment and above the solution throughout the measurements.

### Synthesis of pt NPs by the microwave-citric acid assisted method

A specific amount of Pt(NO_3_)_2_, (Pt loading was 10 wt%), is completely dissolved in distilled water, then the solution pH is adjusted at 2 by using 1 mM HNO_3_ and 1 mM Ca(OH)_2_ aqueous solution. Citric acid is added to the stirred solution in a ratio of 1:2 to the Pt (II) metal ion. Dried banana peels are added in this stage while stirring. The solution is heated in a microwave oven with an operating power of 720 W for 1/2 h; in 30 s cycles (20 s on and 10 s off) till a complete dryness^36–38^. A gel then a foam results, which is ignited by the continues heating. The obtained powder is calcined at 900 °C for 3 h in the muffle furnace.

### Electrochemical cell and measurements

A three-electrodes one-compartment electrochemical cell is used to perform the electrochemical measurements, the auxiliary electrode is a large Pt coil, the reference electrode is a saturated 3 M Ag/AgCl, and the working electrode is a glassy carbon (GC) disc electrode (diameter = 1.6 mm). 10 mg of the prepared Pt NPs are homogeneously distributed in 1 mL of DMF by sonication, then few uLs of the catalyst suspension are casted on GC electrode surface. The electrochemical measurements; cathodic polarization and impedance are performed in 0.1 M H_2_SO_4_ aqueous solution using a Gamry interface 1000 potentiostat. Cathodic polarization is carried out from − 0.5 to − 0.6 V, at a scan rate of 1 mV/s. The experiment was initiated by a stabilization at the open-circuit potential for 30 min, followed by two steps activation; at − 0.4 V for 10 min, and − 0.5 V for 5 min. then, the cathodic polarization is done. The impedance experiment is recorded from 0.1 Hz to 100 kHz, with an amplitude of 5 mV, at three overpotentials; − 50, − 75, and − 100 mV vs. the reference electrode.

### Characterization techniques

The structural characterization of the prepared Pt NPs is done by X-ray diffractogram (XRD, Shimadzu, XRD-7000). The sample morphology is examined by scanning and transmission electron microscopy, (SEM, JEOL JXA-840 A) and (TEM, JEOL JEM 1400). The surface area is calculated by Brunauer-Emmett-Teller (BET) method using N_2_ gas as an adsorbate at 77 K, and done by Nova Touch, Quanta Chrome.

## Conclusion

Highly distributed Pt NPs can be prepared by the microwave-assistant citrate method using green and economic carbon-derived biomass support. The prepared sample exhibits high porosity and uniform distribution over the support, as shown by SEM and TEM photos, and the BET specific surface area of 63.16 m^2^/g. A high electrocatalytic activity for the HER is observed by the potentiodynamic linear polarization and impedance spectroscopy. Higher *i*_*o*_ (5.3 mA/cm^2^) and lower *E*_*a*_ (38.13 kJ/mol) values are calculated for the proposed catalyst, indicates the better performance as compared with those of other Pt- and transition metals-based catalysts. The reaction order is close to unity and the mechanism of the HER on the prepared Pt NPs follows Volmer/Tafel mechanism. Impedance spectra reveal the high catalytic activity by the decrease of the total impedance, pore resistance, and charge-transfer resistance with increasing the applied overpotential.

## Data Availability

Data will be available on request from the corresponding author.
